# Heterogeneous graph neural network for lncRNA-disease association prediction

**DOI:** 10.1038/s41598-022-22447-y

**Published:** 2022-10-20

**Authors:** Hong Shi, Xiaomeng Zhang, Lin Tang, Lin Liu

**Affiliations:** 1 School of Information, Yunan Normal University, Kunming, 650092 China; 2 Key Laboratory of Educational Informatization for Nationalities Ministry of Education, Yunnan Normal University, Kunming, 650092 China

**Keywords:** Computational biology and bioinformatics, Biomarkers, Diseases

## Abstract

Identifying lncRNA-disease associations is conducive to the diagnosis, treatment and prevention of diseases. Due to the expensive and time-consuming methods verified by biological experiments, prediction methods based on computational models have gradually become an important means of lncRNA-disease associations discovery. However, existing methods still have challenges to make full use of network topology information to identify potential associations between lncRNA and disease in multi-source data. In this study, we propose a novel method called HGNNLDA for lncRNA-disease association prediction. First, HGNNLDA constructs a heterogeneous network composed of lncRNA similarity network, lncRNA-disease association network and lncRNA-miRNA association network; Then, on this heterogeneous network, various types of strong correlation neighbors with fixed size are sampled for each node by restart random walk; Next, the embedding information of lncRNA and disease in each lncRNA-disease association pair is obtained by the method of type-based neighbor aggregation and all types combination though heterogeneous graph neural network, in which attention mechanism is introduced considering that different types of neighbors will make different contributions to the prediction of lncRNA-disease association. As a result, the area under the receiver operating characteristic curve (AUC) and the area under the precision-recall curve (AUPR) under fivefold cross-validation (5FCV) are 0.9786 and 0.8891, respectively. Compared with five state-of-art prediction models, HGNNLDA has better prediction performance. In addition, in two types of case studies, it is further verified that our method can effectively predict the potential lncRNA-disease associations, and have ability to predict new diseases without any known lncRNAs.

## Introduction

Long non-coding RNAs (lncRNAs) are non-coding RNAs with more than 200 nt (nucleotides) in length^1^. More and more studies have shown that lncRNAs participates in many important biological processes, including gene transcription, cell differentiation and genetic regulation^2^. Moreover, Complex diseases that seriously endanger human health are also inseparable from the abnormal expression of lncRNAs, including diabetes^3^, cardiovascular diseases^4^, HIV^5^, mental disorders^6^ and some cancers such as lung cancer^7^, breast cancer^8^ and prostate cancer^9^. Therefore, identifying the associations between lncRNAs and diseases contributes to understanding the pathogenesis and principles of the diseases, and also provides help for the diagnosis, treatment and prevention of human disease. However, the traditional biological experiments take up a long time, cost much, and have some blindness, all of which will hinder the research process. In recent years, established lncRNA databases such as LncRNADisease2.0^10^, Lnc2Cancer v2.0^11^, NRED^12^, MNDR^13^, and GeneRIF^14^ have made it possible to develop computational methods for predicting potential lncRNA-disease associations. According to the different ideas of algorithms, the existing methods for predicting lncRNA-disease associations can be broadly classified into two categories. They are the method based on biological networks and machine learning, respectively.

Computational methods based on biological networks often rely on the known associations information between lncRNA and disease to build heterogeneous networks. Then lncRNA-disease association prediction is carried out based on this heterogeneous networks. For example, Sun et al.^15^ proposed a network-based computational model RWRlncD, that known lncRNA-disease association network is used to calculate the lncRNA similarity to predict the disease relevance of lncRNAs. Gu et al.^16^ proposed a model for a random walk on a global network (GrwLDA) that uses random walk in the lncRNA similarity network and the disease similarity network to predict potential lncRNA-disease associations. However, GrwLDA had difficulties in optimizing the model parameters. Wen et al.^17^ proposed the Lap-BiRWRHLDA model, which Laplace normalized the similarity matrix before constructing the lncRNA-disease networks, which integrated the two similarity networks through known lncRNA-disease associations, and then predicted lncRNA-disease associations using a double random walk on this heterogeneous networks. Zhang et al.^18^ propose a model LncRDNetFlow based on a global network framework that integrated multisource networks, including lncRNAs similarity network, proteins interaction network, diseases similarity network, and associations information among heterogeneous nodes. The model was able to predict potential associations information for an isolated disease. Zhao et al.^19^ developed a new random walk method MHRWR based on multisource networks. This method introduced disease-gene network and lncRNA-gene network to build a multi-layer network, so as to extract more potential information. Finally, a multi-layer random walk method was used to predict the associations of lncRNA-disease.

Computational methods based machine learning predict potential associations between lncRNAs and diseases by building lncRNA-disease association models, and train the model to improve accuracy using known lncRNA-disease associations data. Chen et al.^20^ assumed that similar diseases were often associated with functionally similar lncRNAs, and developed a model LRLSLDA based on a semi-supervised learning framework, where LRLSLDA effectively predicted potential lncRNA-disease associations by integrating known lncRNA-disease associations and lncRNA expression profiles. Nonetheless, LRLSLDA had the problem of optimize the model parameters. Subsequently, Chen et al.^21^ proposed a new lncRNA-disease prediction model named LNCSIM. LNCSIM further improved LRLSLDA model by introducing lncRNA-disease prediction similarity score. However, this method still could not solve the problem of parameter selection of semantic contribution factors. Zhao et al.^22^ developed a naive Bayesian-based computational approach that integrated various information of disease-related lncRNAs, including genomic, regulome, transcriptome, which resulted in successfully predicting 707 potential cancer-associated lncRNAs. Lan et al.^23^ proposed a novel computational method that used Katcher means to fuse the lncRNA and disease similarity matrixs of multiple data sources and predicted potential lncRNA-disease associations by the SVM classifier. Sheng et al.^24^ used random walk and convolution autoencoders to obtain new feature distributions and then input them into the model to reveal the potential associations between lncRNAs and diseases.

These two types of approaches still have methodological weaknesses. The methods based on biological network rely heavily on the constructed lncRNA-disease heterogeneous network. When network structure changes, this kind of method can’t effectively deal with it. The problem of the method based on machine learning is how to select the optimal features. Most existing machine learning methods do not take full advantage of the rich topological information contained in heterogeneous networks. To make full use of the lncRNAs and diseases feature information and the local and global information on the lncRNA-disease association data, the graph neural network approach appears in some new studies recently. For example, Xuan et al.^25^ used graph convolution network and convolutional neural network to learn the network structure information and the local network features of lncRNA-disease association pair. Wu et al.^26^ used graph convolutional network (GCN) as encoder to obtain the features of lncRNAs and diseases on the heterogeneous network, and then calculated the interaction score between lncRNA and disease by using the inner product of two potential factor vector. Zhang et al.^27^ utilized meta-paths to represent complex semantic information between entities in the network and introduced attention mechanisms to learn the weights of each neighborhood under the metapath and finally aggregate the potential features they obtained from the GCN model. A graph auto-encoder was leveraged to acquire low-dimensional features, finally used a random forest classifer for lncRNA-disease prediction^28^. Zhao et al.^29^ proposed a deep learning algorithm HGATLDA based on heterogeneous graph attention network. HGATLDA uses graph attention network to learn node embedding from isomorphic and heterogeneous subgraphs. In addition, a computational model based on graph attention network and multilayer perceptron (MLP) was proposed for association prediction^30^. However, these methods directly use graph attention network to extract features, and do not take into account the different number of neighbor nodes and the heterogeneity of node types. PANDA applied a graph convolutional auto-encoders for feature extraction and utilized a neural network to predict LDAs^31^.These graph neural network methods realize the capture and utilization of topological information in heterogeneous networks, but ignore the heterogeneity of nodes and edges in heterogeneous graphs.

Inspired by Zhang et al.^32^, the heterogeneity of structure and content in the heterogeneous graph is considered. We propose a novel method for lncRNA-disease association prediction called HGNNLDA. First, a heterogeneous network is constructed, which is composed of the similar network of lncRNAs, the known lncRNA-disease association network and the known lncRNA-miRNA association network. Then, a fixed-size sampling of strongly correlated neighbors is performed by restart random walk for each lncRNA and disease, and the sampled neighbors are grouped according to the types of nodes. Then, the feature vectors of sampled lncRNA, disease and miRNA are obtained by word2vec. The final embedding information of each lncRNA and disease is extracted by aggregating the sampling neighbors according to types and fusing different types, in which attention mechanism is introduced to indicate the importance of different types of neighbors. Finally, the embedding obtained from above steps of each lncRNA-disease association pair are used as the input of classifier, and the prediction score of association pair is calculated. The experimental results show that the AUC and AUPR values of HGNNLDA under fivefold cross validation (5FCV) are 0.9786 and 0.8891, respectively, which is superior to other state-of-art methods. In addition, two case studies show that HGNNLDA has the ability to predict disease-related lncRNA without any known association.

## Results

### Performance evaluation

We considered 2697 known lncRNA-disease associations as positive samples, but the number of positive samples only account for 2.7$\%$ of the total number of samples, so some previous studies^33–36^ selected negative samples with the same number of positive samples from all unknown association pairs. We followed the same strategy and randomly selected 2697 lncRNA-disease associations from all the unknown lncRNA-disease associations to be the negative samples. After constructing the training set of the model, fivefold cross validation (5FCV) was used to evaluate the prediction performance of HGNNLDA. For 5FCV, the sample set was divided into 5 disjoint subsets on average, among which 4 subsets were utilized to train the model and the remaining subset was utilized for testing in each round. Then, HGNNLDA model trained was used to obtain the score of each test sample. The higher the score, the more likely it is that this lncRNA is related to the disease. Next, all test samples were sorted in descending order according to their scores. On this basis, we calculated the true positive rate (TPR) and false positive rate (FPR), Precision and Recall under different thresholds. The specific calculation is as follows:

1 $$\begin{aligned} \begin{aligned} TPR= & {} \frac{{TP}}{{TP + TN}} \qquad FPR = \frac{{FP}}{{TN + FP}}\\ Precision= & {} \frac{{TP}}{{TP + FP}} \qquad Recall = \frac{{TP}}{{TN + FN}} \end{aligned} \end{aligned}$$

Where TP (true positive) means that positive samples are correctly predicted as positive samples; FN (false negative) indicates that the positive sample is erroneously predicted as a negative sample; FP (false positive) means that the negative sample is erroneously predicted as a positive sample; TN (true negative) means that the negative sample is correctly predicted as a negative sample. Then, the ROC curve was drawn with TPR as the vertical axis and FPR as the horizontal axis, and the area under the ROC curve (AUC value) was used as the performance index to evaluate the prediction performance of the model. If the AUC value is larger, the prediction performance of this model is better. To improve the evaluation of the model performance when the positive and negative samples were seriously unbalanced, we also calculated AUPR value to evaluate the overall performance of the model.

### Comparison with other models

**Figure 1:**
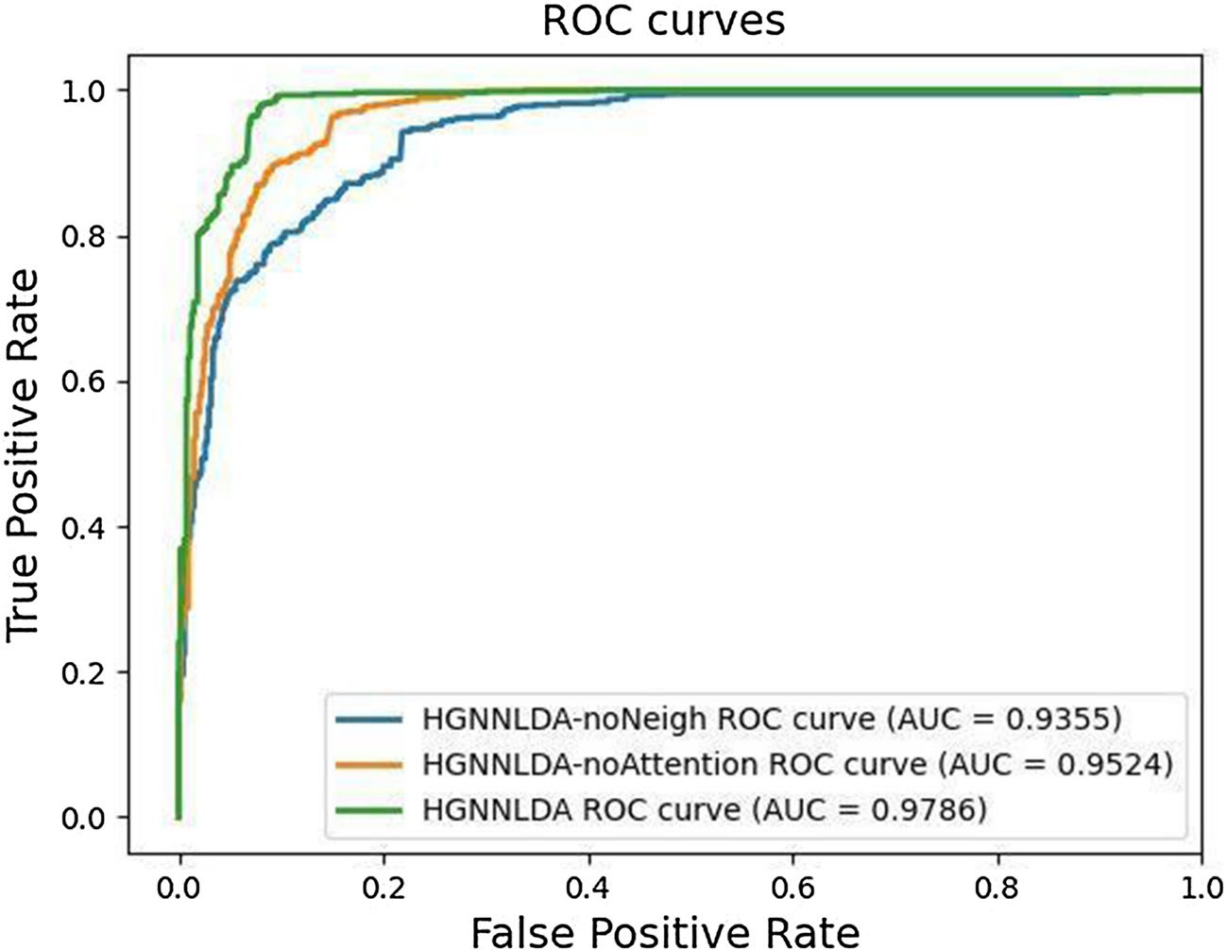
ROC curves of HGNNLDA based on fivefold cross-validation.

In order to further evaluate the prediction performance of HGNNLDA method, we compared it with five state-of-art lncRNA-disease association prediction models, such as SIMCLDA^37^, MFLDA^38^, LDAP^23^, CNNLDA^39^ and GCNLDA^25^. Under the 5FCV, the average AUCs and AUPRs of all lncRNA-disease association prediction models as shown in Table 1. All experimental results of compared models come from Yao et al.’s previous study on lncRNA-disease association prediction, and the results of these models were tested on the same datasets^40^. ROC curve of each cross-validation of HGNNLDA is shown in Fig. 1. Other models didn’t take into account that the neighbor nodes of some nodes may not contain all types of nodes, and assumed that each type of node contributed the same to the prediction of lncRNA-disease association. HGNNLDA solves the problems of other models by restarting random walk and introducing the attention mechanism.

**Table 1:** The mean AUCs and AUPRs of different methods.

Method	AUC	AUPR
SIMCLDA	0.746	0.095
MFLDA	0.626	0.066
LDAP	0.863	0.166
CNNLDA	0.952	0.251
GCNLDA	0.959	0.223
HGNNLDA	0.9786	0.8891

**Figure 2:**
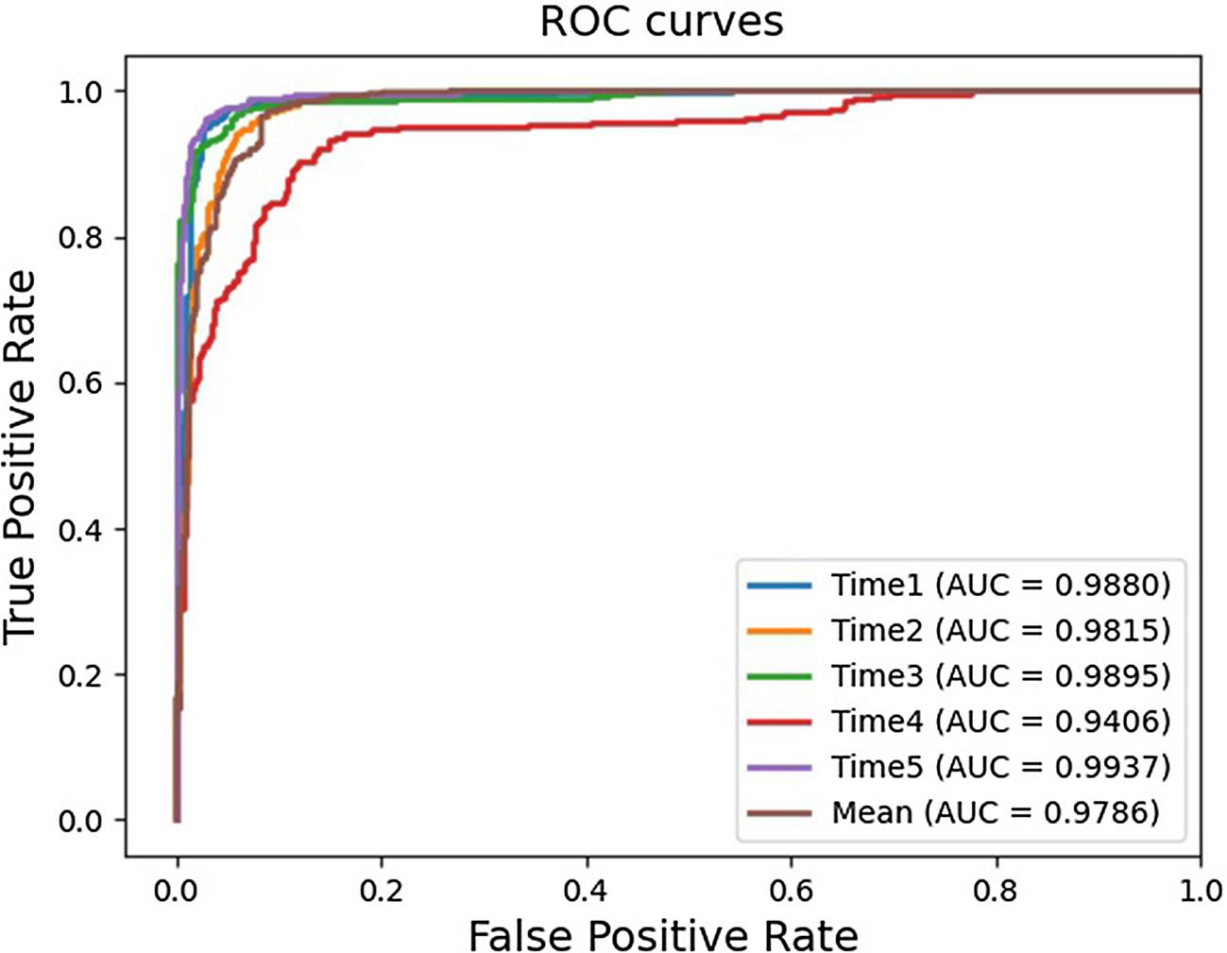
Performance of HGNNLDA and its variants.

**Figure 3:**
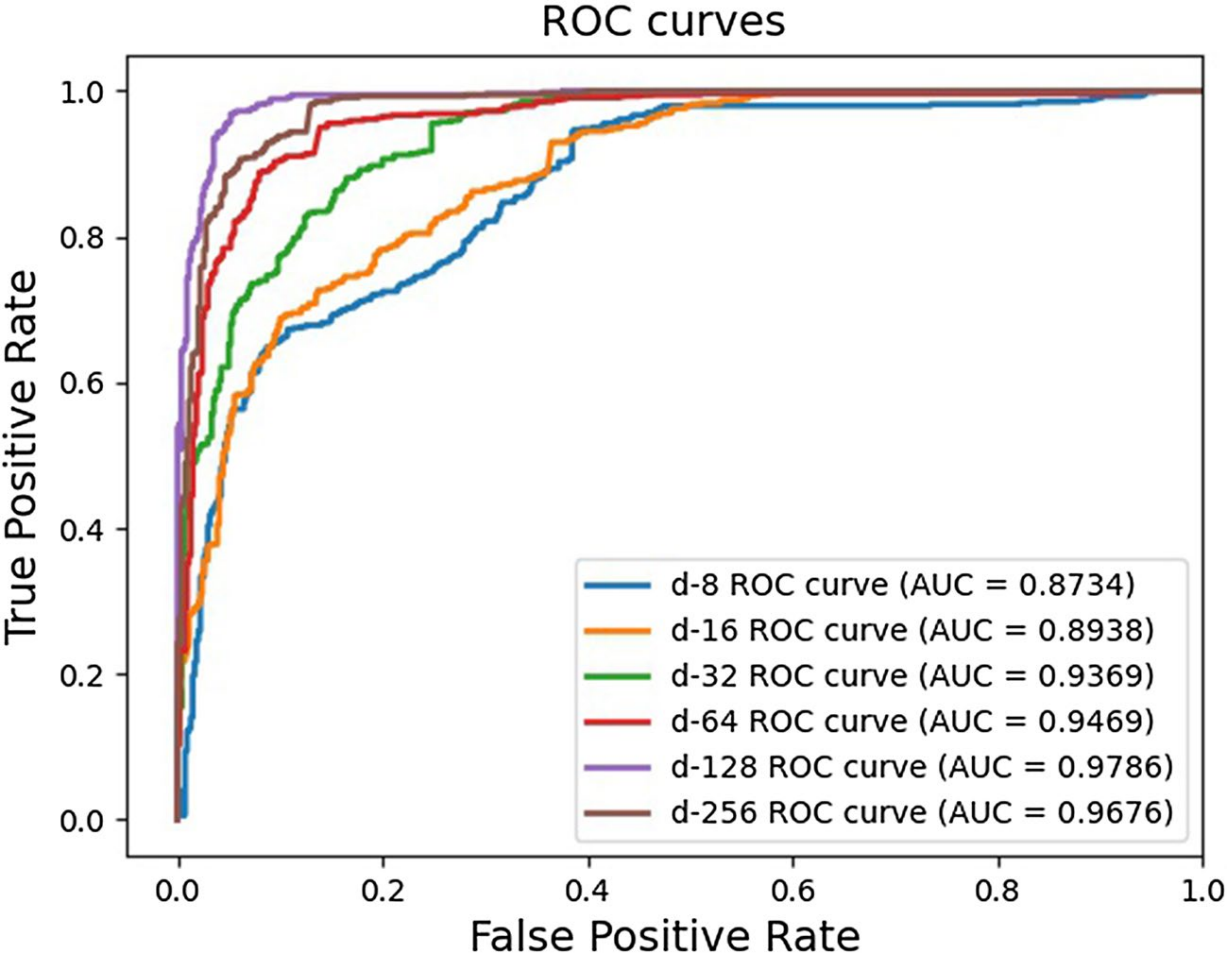
Impact of embedding size.

### Ablation study

To analyze the necessity of each component of our model, we adopt two variants of HGNNLDA (HGNNLDA-noNeigh and HGNNLDA-noAttention) as the comparison method. Specifically, HGNNLDA-noNeigh means that the embedded information of each node is only obtained by word2vec, and the information of any neighboring nodes is not aggregated. HGNNLDA-noAttention uses fully connected neural network instead of attention mechanism to aggregate the embedding of different types of neighbors, which means that different types of neighbor nodes are equally important for the final embedding of lncRNA and disease. Figure 2 shows the average AUC obtained using HGNNLDA and two variant models. HGNNLDA has better performance than HGNNLDA-noNeigh, which indicates that aggregating the information of neighboring nodes can better generate the embedded information of nodes. HGNNLDA gets better results than HGNNLDA-noAttention, which shows that attention mechanism can capture the influence of different types of nodes.

### The effects of embedding size

Embedding size plays an important role in HGNNLDA, which is able to directly affect the performance of the model. In the experiment, we set different embedding dimension d (i.e. 8, 16, 32, 64, 128, 256), and evaluated the prediction performance under different setting. As can be seen from Fig. 3, within a certain range, the larger the embedding dimension, the better the node representation can be learned, and the higher the AUC value. However, when the embedding dimension increase continuously, the AUC value will become stable or slightly worse, which may be caused by over-fitting. Accounting for this factor, the embedding size is set to 128 in this paper.

**Table 2:** The top 10 predicted lncRNAs associated with lung cancer, colon cancer, osteosarcoma.

Rank	LncRNA	Disease	Evidence
1	GAS5	Lung cancer	LncRNAdisease
2	NEG8	Lung cancer	Lnc2Cancer
3	HOOTTIP	Lung cancer	Lnc2Cancer
4	LINC00472	Lung cancer	Lnc2Cancer
5	ZFAS1	Lung cancer	Lnc2Cancer
6	HULC	Lung cancer	Lnc2Cancer
7	BCAR4	Lung cancer	Lnc2Cancer
8	CASC15	Lung cancer	Lnc2Cancer
9	BCYRN1	Lung cancer	Lnc2Cancer
10	GHET1	Lung cancer	Lnc2Cancer
1	UCA1	Colon cancer	LncRNAdisease
2	OIP5-AS1	Colon cancer	Lnc2Cancer
3	HOTTIP	Colon cancer	LncRNAdisease
4	HOTAIR	Colon cancer	LncRNAdisease
5	LINC00319	Colon cancer	Unconfirmed
6	PVT1	Colon cancer	Lnc2Cancer
7	GAS5	Colon cancer	Lnc2Cancer
8	KCNQ10T1	Colon cancer	Lnc2Cancer
9	DANCR	Colon cancer	Lnc2Cancer
10	BANCR	Colon cancer	Unconfirmed
1	NEAT1	Osteosarcoma	Lnc2Cancer
2	XIST	Osteosarcoma	Lnc2Cancer
3	CCAT1	Osteosarcoma	LncRNAdisease
4	EWSAT1	Osteosarcoma	LncRNAdisease
5	AFAP1-AS1	Osteosarcoma	Unconfirmed
6	KCNQ10T1	Osteosarcoma	Lnc2Cancer
7	MIR155HG	Osteosarcoma	Unconfirmed
8	GAS5	Osteosarcoma	Lnc2Cancer
9	PVT1	Osteosarcoma	Lnc2Cancer
10	OIP5-AS1	Osteosarcoma	Lnc2Cancer

**Table 3:** The top 30 predicted lncRNAs associated with breast cancer.

LncRNA (1-15)	Evidence	LncRNA (16-30)	Evidence
H19	Lnc2Cancer	LSINCT5	Lnc2Cancer
HOTTIP	Lnc2Cancer	PVT1	Lnc2Cancer
CDKN2B-AS1	LncRNAdisease	ZFAS1	Lnc2Cancer
AFAP1-AS1	Lnc2Cancer	NCRUPAR	Unconfirmed
KCNQ1OT1	Lnc2Cancer	SOX2-OT	LncRNAdisease
LINC00472	Lnc2Cancer	TP53COR1	Unconfirmed
CASC16	LncRNAdisease	BCAR4	Lnc2Cancer
MALAT1	Lnc2Cancer	NPSR1-AS1	Unconfirmed
NEAT1	Lnc2Cancer	GHET1	Lnc2Cancer
LINC00583	LncRNAdisease	MIR17HG	LncRNAdisease
XIST	Lnc2Cancer	LINC-ROR	Lnc2Cancer
HOTAIR	Lnc2Cancer	NBAT1	Lnc2Cancer
CCAT2	Lnc2Cancer	BANCR	Lnc2Cancer
BCYRN1	LncRNAdisease	HOTAIRM1	Lnc2Cancer
SPRY4-IT1	Lnc2Cancer	DANCR	Lnc2Cancer

### Case studies

To further verify the accuracy and effectiveness of HGNNLDA, we conducted two types of case studies.

For the first type of case study, we applied our proposed method to predict the potential lncRNA-disease associations of three common diseases (lung cancer, colon cancer and osteosarcoma). First, for a specific disease, we regarded all known associations between lncRNAs and diseases as training samples and unknown associations with this disease as candidate samples. Then, we scored all unknown candidate samples of lncRNA-/lung cancer/colon cancer/osteosarcoma, then sorted the scores in descending order and select the top 10 candidate associations related to this disease. The prediction results were verified using two databases (LncRNADisease^10^ database and LncRNA2Cancer^11^ database). Table 2 showed the top 10 results of predicting the potential associations with lung cancer, colon cancer and osteosarcoma, the accuracy reached 100$\%$, 80$\%$ and 80$\%$ respectively. The results showed that our method can effectively predict the potential lncRNA-disease associations.

For the second type of case study, We evaluated the ability of our proposed method to predict the new associations of diseases without any known related lncRNA. We took breast as an example in this case study. First, we set the known associations of breast cancer as unknown associations, and all lncRNAs were considered as candidate lncRNAs. The HGNNLDA was used to score these candidate lncRNAs associated with breast cancer. We found that 27 of the top 30 lncRNA were confirmed by LncRNAdisease database or LncRNA2Cancer database, as shown in Table 3. This result shows that HGNNLDA can effectively predict the potential associations of diseases without any known related lncRNAs.

## Discussion

Identifying associations between lncRNAs and diseases will have a huge impact on our treatment and prevention of some diseases. Therefore, we propose a novel method HGNNLDA to predict the potential associations between lncRNAs and diseases. From the comparison of experimental results, it can be seen that HGNNLDA has superior performance for predicting lncRNA-disease associations. In addition, two types of cases also verify that HGNNLDA has the ability to identify potential lncRNA-disease associations, and can effectively predict new diseases without any known lncRNA.

The reliable performance of HGNNLDA is related to the following factors. First, the model integrates multiple sources of heterogeneous data to build a heterogeneous networks. Second, HGNNLDA gets all types of strong related neighbors of fixed size for each node by restarting random walk, which solves the defect that the direct related neighbors of some nodes are not representative enough. In addition, HGNNLDA is able to capture the strong correlation neighbor features of each node in this heterogeneous network, and fully exploiting the topology information of the heterogeneous network. Finally, HGNNLDA employs the attention mechanism to account for the differential impact of different types of nodes on lncRNA-disease association prediction. To sum up, HGNNLDA makes full use of the complex structural and semantic information of heterogeneous network, so as to achieves good prediction of lncRNA-disease associations.

However, our method still has some limitations. First, the data we use to build heterogeneous networks may contain noise and some outliers. Second, we randomly select the unknown lncRNA-disease association pairs as negative samples for training, which can’t guarantee that the lncRNA and disease in the unknown association pairs are completely unrelated, so it will have some influence on the prediction performance. Therefore, our future research will focus on how to overcome these problems.

## Methods

**Figure 4:**
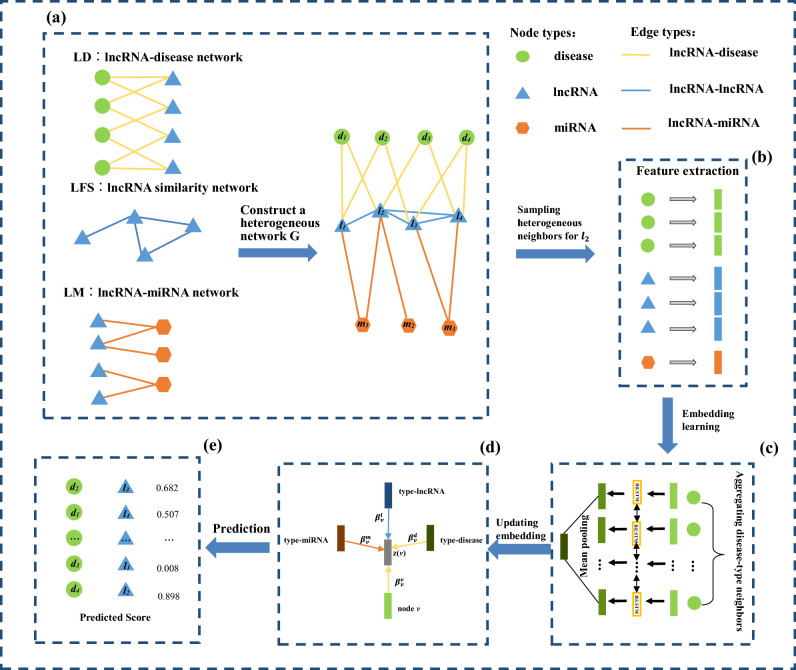
The framework of HGNNLDA.

The general overview of our proposed HGNNLDA framework is shown in Fig. 4, which consists of five key parts: (1) Construction of heterogeneous networks. First we downloaded lncRNA-disease associations, lncRNA-miRNA associations, and calculated the similarity between lncRNAs, and then constructed a heterogeneous network containing the three types of nodes of lncRNA, disease, and miRNA. (2) Sampling strong correlation neighbors and the feature representation of each neighbor. We sampled various types of fixed-sized neighbors for each lncRNA and disease by restart the random walk, and then extract the features of each neighbor node by word2vec. (3) Embedding learning. We used Bi-LSTM to obtain embedding for the three types of neighbors, lncRNA, disease, and miRNA. (4) Updating the node embedding. We introduced the attention mechanism, and aggregated the embedding of three types of neighbors and the embedding of nodes themselves based on the weights obtained. (5) LncRNA-disease association prediction. The embedding of lncRNA and disease were concatenated to get the embedding of lncRNA-disease association pair, then the prediction scores between lncRNA and disease were obtained by using fully connected and softmax layers, eventually optimizde the model by cross-entropy.

### Datasets for lncRNA-disease associations prediction

Studies have shown that lncRNA can interact with the corresponding miRNA and perform biological functions together with miRNAs^41^. Therefore, all useful biological information can be assembled to construct a heterogeneous network including the lncRNA-lncRNA similarity network, the experimentally validated lncRNA-disease association network, and the lncRNA-miRNA association network. The data used in this paper were obtained from the previous study of lncRNA-disease association prediction by Fu et al.^38^. This dataset included 240 lncRNAs, 412 diseases, and 495 miRNAs. Among them, 2,697 verified lncRNA-disease associations are derived from LncRNADisease^10^, Lnc2Cancer^11^ and GeneRIF^14^ databases. In addition, 1002 lncRNA-miRNA associations came from starBase database^42^.

### LncRNA functional similarity network

In this paper, the functional similarity of lncRNA is calculated by the method of Chen et al.^21^. LncRNA similarity is expressed by the similarity of lncRNA related diseases. Suppose that lncRNA $l\left( 1 \right)$ is associated with a group of diseases $D\left( 1 \right) = \left\{ {d\left( {11} \right) ,d\left( {12} \right) , \ldots ,d(1m)} \right\}$, lncRNA $l\left( 2 \right)$ is associated with a group of diseases $D\left( 2 \right) = \left\{ {d\left( {21} \right) ,d\left( {22} \right) , \ldots ,d\left( {2n} \right) } \right\}$. Then the functional similarity between lncRNA $l\left( 1 \right)$ and $l\left( 2 \right)$ is represented by ${S_{l\left( 1 \right) ,l\left( 2 \right) }}$ as follows:

2 $$\begin{aligned}&{S_{l\left( 1 \right) ,l\left( 2 \right) }} = \frac{{\sum \nolimits _{1 \le i \le m} {\mathop {\max }\limits _{1 \le j \le n} \left( {DSS\left( {d(1i} \right) ,d\left( {2j} \right) } \right) } + \sum \nolimits _{1 \le j \le n} {\mathop {\max }\limits _{1 \le i \le m} \left( {DSS\left( {d(2j} \right) ,d\left( {1i} \right) } \right) } }}{{m + n}} \end{aligned}$$

3 $$\begin{aligned}&LFS = \left( {\begin{array}{*{20}{c}} {{S_{l\left( 1 \right) ,l\left( 1 \right) }}}&{} \ldots &{}{{S_{l\left( 1 \right) ,l\left( {240} \right) }}}\\ \vdots &{} \ddots &{} \vdots \\ {{S_{l\left( {240} \right) ,l\left( 1 \right) }}}&{} \cdots &{}{{S_{l\left( {240} \right) ,l\left( {240} \right) }}} \end{array}} \right) \end{aligned}$$

where $DSS\left( {d\left( {1i} \right) ,d\left( {2j} \right) } \right)$ represents the semantic similarity between disease $d\left( {1i} \right)$ and disease $d\left( {2j} \right)$, which adopts the method calculated by Wang et al.^43^; *m* and *n* represent the number of diseases in disease group $D\left( 1 \right)$ and $D\left( 2 \right)$, respectively; *LFS* is a functional similarity matrix of 240 × 240, and 240 represents the number of lncRNAs.

### LncRNA-disease associations and lncRNA-miRNA associations

The datasets includes 2697 experimentally verified lncRNA-disease associations and 1002 experimentally verified lncRNA-miRNA associations^38^. The associations between lncRNAs and diseases are expressed by a 240 × 412 adjacency matrix *LD*, $LD\left( {l\left( i \right) ,l\left( j \right) } \right) = 1$, if lncRNA $l\left( i \right)$ is related to disease $d\left( j \right)$, otherwise it is 0. Similarly, the associations between lncRNAs and miRNAs are represented by an adjacency matrix *LM* of 240 × 495, $LM\left( {l\left( i \right) ,m\left( j \right) } \right) = 1$, if lncRNA $l\left( i \right)$ is related to miRNA $m\left( j \right)$, otherwise it is 0.

### Heterogeneous network construction

As shown in Fig. 4a, we construct a heterogeneous network based on lncRNA functional similarity *LFS*, lncRNA-disease association network *LD* and lncRNA-miRNA association network *LM*. Heterogeneous networks can be expressed as:

4 $$\begin{aligned} G = \left( {N,E,NT,ET} \right) \end{aligned}$$

where *N* represents the node set, which contains three types of nodes, namely $NT = \left\{ {\ln cRNA,disease,miRNA} \right\}$, *E* represents the edge set, which contains three types of edges, namely $ET = \left\{ {\ln cRNA{\text{-}}disease,\ln cRNA{\text{-}} \ln cRNA,\ln cRNA{\text{-}}miRNA} \right\}$.

### Sampling heterogeneous neighbors with restart random walk

In heterogeneous networks, the neighbors of many nodes cannot include all types of nodes, and the number of neighbor nodes will vary^32^. For example, in Fig. 4a, no disease node is directly connected to the miRNA node, and ${d_1}$ has two neighbor nodes, while ${l_2}$ has seven neighbor nodes. Therefore, to make full use of the information of heterogeneous networks, we introduced restart random walk (*RRW*) to sample three types of strongly correlated neighbors for each node. The sampling operation of *RRW* in lncRNA-disease heterogeneous network includes two steps:

- Selecting fixed size sampling length for *RRW*. Starting random walk from node $v \in N$, return to the starting node with probability *p* or iteratively move to the neighbor of the current node, where the probability *q* controls whether the walk is depth first select or breadth first select. When $q > 1$, random walk tends to give priority to breadth; when $q < 1$, random walk tends to give priority to depth. *RRW* runs until a fixed number of nodes are successfully collected, and the sampled nodes are denoted as $\left| {RRW\left( v \right) } \right|$. Moreover, the number of different types of nodes in $\left| {RRW\left( v \right) } \right|$ is constrained, which ensure that all types of nodes are sampled.
- Grouping neighbor nodes of lncRNA, disease and miRNA-type. For each node type *t*, the top ${k_t}$ nodes are selected based on the frequency of occurrence, and take them as the set of *t*-type correlated neighbors of node *v*.

In this way, three types of neighbors can be collected for each node, and classification by type is conducive to subsequently learn embedding of type.

### Embedding learning

Word2vec is a word embedding technology proposed by Mikolov et al.^44^, it can provide a vectorized representation for each word or sentence while preserving semantic and contextual integrity. In the last step, each node obtained a fixed size sampling sequence by using the strategy based on *RRW*. Therefore, Word2vec can be used to get the embedding of each node. Next, to obtain the embedding of type, we can aggregate all the same type neighbors after sampling by using Bi-LSTM^45^. For example, we can express disease-type neighbors of node $v \in N$ in the heterogeneous network as ${N_d}\left( v \right)$. Next, the embedding of each disease-type neighbor node is obtained by Word2vec^44^, as shown in Fig. 4b. Then we utilize Bi-LSTM to aggregate the embeddings of all disease-type neighbors, as shown in Fig. 4c. In the process of aggregating all lncRNA-type nodes, disease-type nodes and miRNA-type nodes, different Bi-LSTM are used to distinguish them. Bi-LSTM consists of a forward LSTM layer and a backward LSTM layer. The main structure of LSTM layer can be expressed as follows:

5 $$\begin{aligned} \begin{aligned} {i_s} & = \sigma \left( {{w_s} \cdot f\left( s \right) + {h_{s - 1}} \cdot {w_{s'}} + {b_f}} \right) \\ {f_s} &= \sigma \left( {{w_f} \cdot f\left( s \right) + {h_{s - 1}} \cdot {w_{f'}} + {b_f}} \right) \\ {o_s} &= \sigma \left( {{w_o} \cdot f\left( s \right) + {h_{s - 1}} \cdot {w_{o'}} + {b_o}} \right) \\ {\tilde{c}_s} &= \sigma \left( {{w_c} \cdot f\left( s \right) + {h_{s - 1}} \cdot {w_{c'}} + {b_c}} \right) \\ {c_s} &= {i_s} \otimes {\tilde{c}_s} + {f_s} \otimes {c_{s - 1}}\\ {h_s} &= {o_s} \otimes \tanh \left( {{c_s}} \right) \end{aligned} \end{aligned}$$

Where $\sigma$ is sigmoid activation function; *i*, *f*, *o* and *c* represent input gate vector, forget gate vector, output gate vector and memory unit respectively; ${h_s}$ represents the output hidden vector by *s*-th node; *w* and *b* represent learnable parameters; $\otimes$ represents dot product operation. Two different middle layer representations can be obtained through calculation. Then, after splicing the two middle layers, the general embedding of all disease-type neighbor nodes of node *v* can be obtained through the average pool layer, as shown follow:

6 $$\begin{aligned} \begin{aligned} \overrightarrow{{h_s}} & = LST{M_d}\left( {\overrightarrow{{h_{s - 1}}} ,f\left( s \right) } \right) \\ \overleftarrow{{h_s}} & = LST{M_d}\left( {\overleftarrow{{h_s}} ,f\left( s \right) } \right) \\ {f^d}\left( v \right) & = \frac{{\sum \nolimits _{s \in {N_d}\left( v \right) } {\overrightarrow{{h_s}} \oplus \overleftarrow{{h_s}} } }}{{\left| {{N_d}\left( v \right) } \right| }} \end{aligned} \end{aligned}$$

Where ${f^d}\left( v \right) \in {\mathrm{{R}}^{d \times 1}}$ is the general embedding of all disease-type neighbors of node *v*; $\overrightarrow{{h_s}}$ and $\overleftarrow{{h_s}}$ represent the forward and backward LSTM representations of *s* node respectively; the symbol $\oplus$ indicates the connection operation.

### Updating the node embedding with attention mechanism

In the previous step, the general embedding of lncRNA-type, disease-type and miRNA-type will be generated. Different types of neighbors will have different influences on the final embedding of node *v*^32^, for example, nodes of lncRNA, disease-type usually play a more important role in the prediction of lncRNA-disease association. So as to combine lncRNA-type, disease-type and miRNA-type general embeddings with node *v* embedding, we introduce the attention mechanism^46^. First, the importance of each type is learned, and then all heterogeneous types of nodes(including node *v* itself ) are aggregated to form the final embedding of node *v*. For any $t \in N\left( v \right)$, $N\left( v \right) = \left\{ {v \cup NT} \right\}$, the importance $\beta _v^t$ of *t*-type relative to node *v* is expressed as:

7 $$\begin{aligned} \begin{aligned} \beta _v^t = \frac{{\exp \left( {\sigma \left( {{\mathrm{{q}}^\mathrm{{T}}}\left[ {f\left( v \right) \parallel {f^t}\left( v \right) } \right] } \right) } \right) }}{{\sum \nolimits _{k \in N\left( v \right) } {\left( {\exp (\sigma \left( {{\mathrm{{q}}^\mathrm{{T}}}\left[ {f\left( v \right) \parallel {f^k}\left( v \right) } \right] } \right) } \right) } }} \end{aligned} \end{aligned}$$

Where $\sigma$ is *ReLU* activation function; ${\mathrm{{q}}^\mathrm{{T}}} \in {\mathrm{{R}}^{2d \times 1}}$ represents the attention vector; *f*(*v*) is that embedding of *v* obtained by word2vec; ${f^t}\left( v \right)$ is a general embedding based on *t*-type aggregating; $\parallel$ indicates the connection operation; ${f^k}\left( v \right) = f\left( v \right)$ when *k* equals *v*. Then, the final embedding of node *v* can be aggregated by various types of embedding based on the corresponding importance coefficient. The details are as follows:

8 $$\begin{aligned} \begin{aligned} z\left( v \right) = \sigma \left( {\sum \nolimits _{k \in N\left( v \right) } {\beta _v^t{f^k}\left( v \right) } } \right) \end{aligned} \end{aligned}$$

Where $z\left( v \right) \in {\mathrm{{R}}^{d \times 1}}$ represents the final embedding. To better understand the aggregation process of various types of nodes, explanation is shown in Fig. 4d.

### LncRNA-disease association prediction

The final embedding of lncRNA ${l_i}$ and the final embedding of disease ${d_j}$ are spliced to constitute the vector representation ${x_{i,j}}\in {\mathrm{{R}}^{2d \times 1}}$ of the association pair ${l_i} - {d_j}$:

9 $$\begin{aligned} {x_{i,j}} = z\left( {{l_i}} \right) \otimes z\left( {{d_j}} \right) \end{aligned}$$

Where $\otimes$ represents splicing operation. Then, each positive sample (there is an association between lncRNA and disease) is marked as 1, and each negative sample (there is no association between lncRNA and disease) is marked as 0. Then, we provide the embedding of the association pair ${l_i} - {d_j}$ to the fully connected layer and the *softmax* layer, and the score of association ${s_{i,j}} \in \left[ {0,1} \right]$ between lncRNA ${l_i}$ and disease ${d_j}$ is obtained. The specific ${s_{i,j}}$ is expressed as follows:

10 $$\begin{aligned} {s_{i,j}} = softmax\left( {W{x_{i,j}} + b} \right) \end{aligned}$$

Where $W \in {\mathrm{{R}}^{2 \times 2d}}$ is the parameter of the full connection layer and *b* is the bias; the larger the score of ${s_{i,j}}$, the greater the possibility of association between lncRNA ${l_i}$ and disease ${d_j}$. In our model, the cross-entropy loss between prediction and real association is defined as follows:

11 $$\begin{aligned} Loss = - \sum \limits _{i = 1}^T {{y_i}\log {s_i}} \end{aligned}$$

Where *T* is the number of training samples; ${s_i}$ is the score of the association between lncRNA and disease of training sample; ${y_i}$ is the label of real association between lncRNA and disease.

## Data Availability

The original datasets of our study was download from another lncRNA-disease association prediction study, the orginal datasets were available at https://github.com/ydkvictory/RFLDA. The processed data along with codes are available at https://github.com/hongshi940/HGNNLDA.
